# A Total Economic Valuation of Wetland Ecosystem Services: An Evidence from Jagadishpur Ramsar Site, Nepal

**DOI:** 10.1155/2016/2605609

**Published:** 2016-10-17

**Authors:** Sony Baral, Bijendra Basnyat, Rajendra Khanal, Kalyan Gauli

**Affiliations:** ^1^International Union for Conservation of Nature, Kathmandu, Nepal; ^2^Institute of Forestry, Pokhara, Nepal; ^3^Multi Stakeholder Forestry Programme, Lalitpur, Nepal

## Abstract

Wetlands are the most productive ecosystem and provide wide arrays of wetland ecosystems (goods and services) to the local communities in particular and global communities in general. However, management of the wetland often does not remain priority and recognized as the unproductive waste land mainly due to poor realization of the economic value of the wetlands. Taking this into account, the study estimated the total economic value of the Jagadishpur Reservoir taking into account direct, indirect, and nonuse value. The study prioritized six major values of the reservoir which include wetland goods consumption, tourism, irrigation, carbon sequestration, biodiversity conservation, and conservation for future use (existence and option value). The study used market and nonmarket based valuation techniques to estimate total economic value of the reservoir. Household survey, focus group discussions, and interaction with the tourism entrepreneurs and district stakeholders were carried out to collect information. The study estimated the total annual economic value of the reservoir as NRs 94.5 million, where option/existence value remains main contributor followed by direct use value such as wetland goods and tourism and indirect use value, for example, carbon sequestration, biodiversity conservation, and irrigation. The study reveals that the local communities gave high importance to the future use value and are willing to make investment for conservation and restoration of reservoir given its conservation significance.

## 1. Introduction

Wetlands are amongst the most diverse and productive ecosystems of the world and are of immense socioeconomic importance [1]. Wetlands provide wide arrays of goods and services to the local communities and also the people living outside the periphery [2]. Wetlands provide numerous goods and services to society, supporting millions of people around the world. Indeed, the goods and services help life support system, conserve biological diversity, and act as safety net and an environmental insurance against the impacts of climate change and ecosystem degradation [3]. Ramachandra et al. found that anthropogenic activities impact physical, biological, and chemical processes of wetlands, which impair the ecosystem functioning causing decline and degradation of ecosystem services and also economic value of wetlands. The global values of direct goods from wetland and associated ecosystems services have been estimated at US$14 trillion annually [4]. They provide food, fodder, fuel, and water for domestic, irrigation, and industrial purposes. They are critical for contributing to poverty reduction. Furthermore, it also serves as the kidney of the landscape because of functions they perform in the hydrological and chemical cycles [5]. Despite their significant role in maintaining the healthy ecosystem and contribution to the local livelihoods of the people, wetlands are under threat due to degradation of catchments and water diversion leading to changes in water regimes. Many parts of the world have experienced loss or degradation of wetlands mainly due to agricultural use, urbanization, excessive exploitation by local populations, and poor planned developmental activities [6]. Globally, several studies on economic valuation of wetlands have been carried out; however very few studies which focus on the total economic contribution of wetlands have been carried out in which the annual value of goods and services from wetland was estimated to be second highest, US$14785/ha based on the assessment of 17 ecosystems services in 16 biomes, which emphasise on social welfares [7]. Numerous studies suggested that there is not clear definition of wetlands goods and services and the real economic value of services and their importance to social welfare and local and national economy, being the main reason for poor management of such resources [3, 4, 8].

Overall, Nepal hosts great wetlands diversity covering a total of 743,563 ha, which represents 5% of the total landmass of the country [9]. Nepal's wetlands include different types that range from areas of permanently flowing rivers to areas of seasonal streams, lowland oxbow lakes, high altitude glacial lakes, swamps and marshes, paddy fields, reservoirs, and ponds. Nepal's wetlands support a wide spectrum of nationally and globally important biodiversity and harbor 42 globally threatened species [10]. In addition to providing habitat for several species of wildlife, their role in sustaining people's livelihoods is crucial. By taking this in mind several studies undertaken for the other wetlands of Nepal have shown the importance of wetland conservation and emphasized increasing investment according to the value of wetland resources [11]. However, being the second biggest manmade wetland “Jagadishpur” in Asia has not been getting sufficient attention in the management and conservation. This has resulted in their continuous loss and threats of the wetland resources.

Sustainable management of wetland is crucial for the welfare of local communities. However, the management of the wetland often does not remain priority and recognized as the unproductive waste land, mainly due to poor realization of the economic value of the wetlands. They offer provisioning, regulating, cultural, and supporting services that generate economic value from their direct, indirect, or potential use [12]. There is an urgent need for a balance to be struck between wetland conservation, sustainable utilization, and wetland conversion. The economic values of nonmarket goods and services should be measured in monetary terms to recognize true economic contribution, maximize long term benefits, and increased investment in conservation [13]. Hence, from the point of view of both ecological and economics perspective, the significance of wetland and the participation of stakeholders for preservation is crucial. The main objective of this paper is to evaluate the total economic valuation of the major goods and services of Jagadishpur Ramsar site.

## 2. Methodology

The study adopted a total economic valuation approach for identifying array of values that are attributed to JRRS. Total economic value (TEV) is a well-established and useful framework for identifying the various values associated with protected areas [14]. It consists of direct use, indirect use, and nonuse values. This approach helps to avoid double counting of ecosystem functions, intermediate services, and final services [11]. Understanding of the economic value of the ecosystem services is very important for informed decision making [15]. TEV also provides policy guideline for allocation of scarce public resources for the conservation and development in light of growing demand of both environmental services.

### 2.1. Study Site

**Figure 1:**
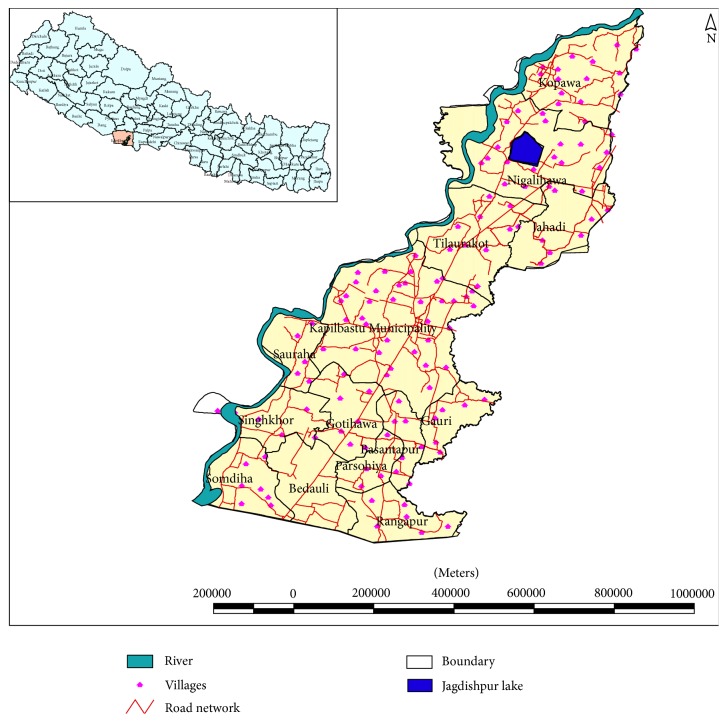
Study site.

The Jagadishpur Reservoir catchment area covers 196 sq. km. area of 13 local government units [16]. The reservoir is one of the largest manmade wetlands, constructed in 1979 for irrigation. The reservoir with an area of 118 ha has the capacity to store 4.7 million cubic meters of water, which can irrigate 6070 ha of farm lands (Figure 1). The reservoir was declared as a Ramsar site in 2003, in recognition of the fact that it supports vulnerable, endangered, and critically endangered species as well as threatened ecological communities [9]. It is surrounded by cultivated land, canals, and small ponds.

The reservoir and its surrounding area are rich in biodiversity. It is one of the important bird areas of Nepal. It provides a home for many species of migratory waterfowl, including endangered species like* sarus crane*. Of the 871 species of birds recorded in the country, 168 species belonging to 42 families are reported in the JRRS. Furthermore, 28 species of bird are either globally or nationally threatened or included in the CITES Appendices and/or IUCN Red List [16].

It supports four percent of the Asian population of Ferruginous Duck and one percent of the Lesser Whistling Duck population found in Nepal [9]. A total of 295 species of fauna are reported in the area; 19 are nationally threatened and 48 are included in the IUCN Red List while 37 species are included in the CITES Appendices [16].

Water User Association of Jagadishpur is managing the irrigation system in partnership with the government. About 17,390 households, with populations of 54,358 are dependent on reservoir for irrigation, fish, foods, and recreational use [16]. Likewise, JRRS is being also used for grazing, forests productions collection, and household purposes.

### 2.2. Study Methods

The study adopted the following six sequential steps for total economic valuation of the reservoir (Table 1). The study identified four major stakeholders who have stake on reservoir, namely, local communities, business entrepreneurs/restaurant owners, community based organizations, and government line agencies. The study first listed out different use and nonuse value of the reservoir based on consultations with the stakeholders. Each group of stakeholders was requested to prioritize different categories or types of use and nonuse value of the reservoir. The score was then summed up to identify top five types of value, representing all three categories of the value. After prioritization, earlier valuation studies conducted in Nepal and elsewhere were reviewed to select the appropriate valuation methods and field survey was executed to collect information. The study then quantified the total value economic value of the reservoir.

The total economic value of the reservoir takes into account the direct use, indirect use, and nonuse value. The study prioritized two direct use values, namely, wetland goods consumption and tourism, and three indirect use values, namely, carbon sequestration, water supply, and biodiversity conservation (Table 2). The nonuse value includes conservation/restoration of the reservoir for future use. Overall, we calculated values for six categories of ecosystem goods and services, condensed from a previously published [1] with eight categories and [4] with 17 categories.

*Wetland Goods.* The study conducted household survey to estimate (a) total value of the wetland goods consumed by the households and (b) willingness to pay for conservation and management of the reservoir.

The study followed a stratified random sampling method, where study area was divided into three clusters based on distance from the reservoir, namely,* adjoining area* (within 5 km of reservoir),* nearby area *(between 5 and 10 km from reservoir), and* distant area* (above 10 km from reservoirs) and surveyed households based on population probability to size. The study surveyed a total 384 households, which were statistically representative. Furthermore, focus group discussions, stakeholders' consultations, key informant interview, and observations were carried out to complement survey findings. Household survey was analyzed based on the above strata to estimate total value of wetland goods consumption.

*Tourism.* Total value of tourist earning can be used to estimate value of tourism services [17]. The study used total earning from tourists visiting reservoir to estimate value of recreational services. The study surveyed 15 hotels/restaurants which are operating around the reservoir along with 10 tour operators such as local bus drivers, taxi drivers, and travel agents to estimate total expenditure incurred by visitors' tourism related activities. The expenditure was estimated while considering duration of stay, food and accommodation expenses, recreational expenses (boating), and cost of travel.

*Irrigation.* The reservoir is used for the irrigation purposes, especially for winter crop cultivation. Each farmer is paying irrigation service fee for the maintenance of the reservoir and distribution of the water. The study takes into account water use fee paid by the farmers to estimate water use value of the reservoir.

*Biodiversity Conservation.* Revealed price is one of the best indicators of the prices of goods and services [13]. Highly valued goods and services are allocated higher resources for their conservation and vice versa. Hence, a fund allocated by national or international conservation organizations for conservation of biodiversity hot-spots/protected area is considered as a proxy value of biodiversity [18, 19]. The study used financial and programmatic support provided by national government and conservation partners for protecting the biodiversity for estimating value of biodiversity services. The studies do not take into account investment of government organization for repair and maintenance of the irrigation canal.

*Carbon Sequestration.* The study followed benefit transfer methods to estimate the carbon sequestration value of the reservoir. The forests data was obtained from the IUCN's land use and land cover study [16]. Forest carbon sequestration rate is estimated at 1.38 tCha^−1^ yr^−1^ in Chitwan, Tarai forests of Nepal [20]. Likewise, wetland carbon sequestration rate of the wetland was 1.30 g-Cm^−2^ year^−1^ in tropical/subtropical wetlands [21]. The carbon value was then obtained based on prevailing market price of the carbon.

*Future Use.* Option or future use values refer to the value people assign to the resources in the expectation that they would be a source of various biological and other resources in the future which are yet to be explored. Contingent valuation method estimates willingness to pay (WTP) for the conservation of a resource. WTP is widely used in Nepal and elsewhere [13, 22, 23] for estimation of option and existence value. CVM uses a survey instrument to measure individuals' maximum WTP in a hypothetical market.

The surveyed households were asked about the amount, which they are willing to contribute as cash and voluntary labor for the conservation of the reservoir using a bidding game. Attempts were made to create a situation in the bidding game in such a way that respondent feels that they would really have to contribute the amount in either cash or voluntary labor, which they committed to at the time of survey very soon such that they decide with perfect economic rationality rather than being guided by altruistic motives.

## 3. Results

### 3.1. Prioritization of Wetland Goods and Services

The study consulted with the stakeholders, especially wetland users, government officials, and local government and mapped different goods and services offered by the stakeholders. After listing of the goods and services, they were asked to give score of each services, not exceeding 100 in total. This includes fish, irrigation, edible foods, wildlife parts, grazing, ground water recharge, biodiversity conservation, carbon sequestration, tourism, irrigation, religious and cultural value, livestock bathing, wild edible food, and roofing materials. Of the above categories, the study prioritized six major values of the reservoirs, for total economic valuation, representing all three categories of the value (Table 3). The study included major wetland goods such as fish, tortoise, edible food drift wood under the wetland goods, since stakeholders did not prioritize goods consumed from reservoir.

### 3.2. Value of Wetland Goods and Services

#### 3.2.1. Wetland Goods

The market price method was used for the estimating direct use value of wetland goods by the households. The major wetland goods that are consumed by local communities include fish, turtles, crabs, birds, edible plants, fruits, grasses, and thatches. These goods are obtained through purchase or self-collection. The survey showed that more than one-fourth of households (27.1%) have consumed at least one of the wetland goods, ranging from 44.1% in the nearby area to 16.9% in the distant area. Nearly one-fifth of the households have consumed fish, varying from 16.2% in distant area to 43.4% in nearby area. The consumption of the other wetland goods is virtually nonexistent in the study area (Table 4).

The study computed the average value of the wetland goods consumed in the area based on estimation of the market price of each of the goods. Each household consumed wetland goods of NRs 966 (around US$10) per year varying from Rs 1640 in nearby area to Rs 500 in distant area. The total value of wetland goods consumed from the reservoir would reach about NRs 16.9 million, based on a simple extrapolation (Table 5).

Fish are the main goods consumed from the wetlands, contributing to more than 95% of the total value of the wetland goods (Table 6). The contribution of the other goods is almost negligible.

#### 3.2.2. Tourism

There is no formal recording system for visitors. Nevertheless, about 150 to 200 international and about 10,000 to 12,000 domestic visitors make visit to the Jagadishpur area every year [16]. Of the international visitors nearly two-thirds are Indian. As there are limited tourism facilities and services at the Jagadishpur, hence most of the visitors come for nature walk and enjoy local fishes. Likewise, there were no facilities available for accommodation. Hence, average expenditure was estimated based on expenses on food and cost of travel (Table 7). The cost of travel only includes travel cost from major cities such as Lubmini, Bhairwa, and Butwol to reach the reservoir since almost all visitors visit the reservoir, when they came to visit nearby religious places [16]. Foreign, SAARC, and Nepalese visitors made an average expenditure of NRs 1270, Rs 1823, and 874, respectively, during each visit. The total value of the recreational service is estimated about NRs 9.1 million.

#### 3.2.3. Irrigation

Water is mainly used for irrigation, buffalo bathing, and washing clothes. Farmers mostly used water for irrigating summer and winter crops, such as paddy, wheat, maize, and vegetables. The reservoir can irrigate 6070 ha of farm lands in each season. Each farmer is required to pay NRs 150 per ha per season, that is, NRs 300 per year per ha for using water for irrigation. This would result in irrigation value of NRs 1.8 million per year (Table 8).

#### 3.2.4. Biodiversity Services

The reservoir is rich in biological resources and has high faunal diversity. A total of 68 species are recorded at the wetland sites, of which four are submerged, 19 are emergent, and 13 are floating plant species, while the rest are terrestrial plants. Likewise, 43 species of fish, 52 species of herpetofauna, 168 species of birds, and 32 mammal species are recorded in the area [16]. Biodiversity service is estimated based on government expenditure together with direct and indirect financial supports from conservation partners for conserving natural heritage and biodiversity [13].

District Forest Office, District Development Committee, and District Soil and Water Conservation Office are the major government agencies which are implementing projects and programmes for biodiversity conservation whereas conservation partner, especially International Union for Conservation of Nature, is implementing Wetland for the Future Project focusing on wetland restorations and livelihoods improvement. Government made an annual expenditure of NRs 800,000 for biodiversity conservation and wetland restoration while expenditure of conservation partners was NRs 10.4 million [16] in 2015. Hence, total value of the biodiversity services is estimated at NRs 11.2 million.

#### 3.2.5. Carbon Sequestration

This study also estimated annual carbon sequestration of both wetland and forests (Table 9). The fresh water stored annually 1.3 tCha^−1^ yr^−1^ [21] whereas the forests sequestrate carbon at rate of 1.38 tCha^−1^ yr^−1^ in Chitwan district [20]. The mean of the transactions was US$2.9 per ton of CO_2_ in 2009 [24]. This is equivalent to US$10.64 or NRs 1064 per ton of carbon (1 ton of carbon = 3.67 tons or CO_2_). The wetland ecosystem stored nearly three times higher carbon than forests ecosystem. The total carbon stock value of the reservoir is NRs 1.0 million per year.

#### 3.2.6. Future Use Value

The study used contingent valuation method (CVM) to quantify nonuse/future use value of wetland resources. CVM directly elucidates people's views to determine how much they might be willing to pay for a resource or service.  Table 10 presents number of HHs willing to pay for conservation and management of the JRRS. Of the total households surveyed, nearly two-thirds are willing to pay for conservation of the reservoir. Those households who are willing to pay are interested to pay both in cash and in kind. All the households are willing to contribute free labor for protection of the reservoir as well as contribute in cash.

Table 11 presents average household willingness to pay in cash and kind for the conservation and restoration of the reservoir. On average, each household is willing to pay NRs 539 per year in cash and willing to provide voluntary labor of 5.9 days per year, or the equivalent of NRs 2597 per year, calculated by local wage rates. Willingness to contribute in cash as well as in labor was high in adjoining area of the reservoir while it was less in the distant area. The nonuse value would result in NRs 54.5 million, when simple extrapolation is made with number of households in the reservoir.

### 3.3. Total Economic Value

As shown in Table 12, total economic value (TEV) of reservoir is estimated as NRs 94.5 million. Of the different value, future use value (option or existence value) contributes more than half of the value of the reservoir followed by the direct use value (wetland goods and recreation) and non-use value (carbon, biodiversity and water use). High nonuse option value shows the importance of the reservoir in conservation and protecting for the future needs.

Total economic value of the wetland was divided by the total households benefiting from reservoir (17,390 households) to compute value of wetland for each household while value was divided by area of the reservoir (18,506 ha) to compute value by unit area ha. The total value of wetlands for each HH is NRs 5439 while it is NRs 4825/ha in terms of area.

## 4. Discussion

Value of wetland goods consumed by each household in the reservoir per household was NRs 973, which is far below the value of the wetland goods, consumed in the Ghodaghodi lake of Nepal. Lamsal et al. [25] estimated that each household extracted lake resources at an annual worth of NRs 4379. These high difference are mainly because we do not take into account the value of forest goods consumed from JRRS area while Lamsal et al. [25] included the fishes, firewood, timber, and fodder. Of the total wetland goods consumed in the Ghodaghodi lake complex, forest goods contributed more nearly ninety percent of the total wetland goods [25].

The average willingness to pay of domestic, South Asian country, and foreign visitors in the Chitwan national park and buffer zone was estimated at NRs 3370, NRs 6960, and NRs 7500, respectively [26], while tourism expenditure of visitors estimated at Rs 7,667, NRs 16,120, and NRs 23,173 for Bardia National Park for domestic, South Asian country, and foreign visitors [13]. The value of tourism appears far below in the reservoir compared to two protected areas. This is mainly because of availability of the limited tourism facilities and services in the reservoir. Local communities receive little or no benefit from tourists apart from a few hotels where tourists mostly consume local food [16].

The study estimated that willingness of the households is estimated to be NRs 3135 in the Jagadishpur Reservoir, which is far below the average willingness of a household residing in the vicinity of Koshi Tappu Wildlife Reserve which is estimated to be NRs 23,800 [27]. The low willingness pay in the reservoir is mainly because of less conservation awareness of the people, since the reserve was the first Ramsar site of the country.

The total value of wetland is estimated at NRs 973 for each household, which is almost 10 times lower than wetland value estimated by [11] in Koshi Tappu Wildlife Reserve which is around NRs 10,000 for each household. The high difference in value is mostly because of limited use of provisioning services by the households. Likewise, the Koshi Tappu Wildlife Reserve is used for commercial fishing while it is not allowed in reserve. Likewise, differences in wetland goods and services consumed from reservoir also affected wetland value.

The nonuse value accounted for more than half of the wetland value in the reservoir, which is different than other studies conducted in reservoir. In Ghodaghodi lake complex, the wetland goods contributed to more than ninety percent of total value [25] of provisioning services such as fish and forest products, while they accounted for about 85% of Koshi Tappu Wildlife Reserve [16]. This reveals that people of the Jagadishpur give conservation and protection of the reservoir high importance for future use, while people gave wetland goods in other Ramsar sites high importance.

## 5. Conclusion

The Jagadishpur Reservoir provides a wide range of goods and services to the local communities. Of the different 24 types of use and nonuse value of the reservoir, the stakeholders prioritized six values, which include wetland goods, irrigation, carbon sequestration, biodiversity conservation, tourism, and future use value. The total value of the reservoir is NRs 94.5 million per year or Rs 4825 per year per ha. Of the total economic value, nonuse value contributes to more than half of the total value of the reservoir followed by direct use and indirect use value. This reveals that local community has given conservation and restoration of the wetlands high importance and this has not only increased their local pride, but also made them want to conserve reservoir for future generation. Majority of respondents were willing to contribute either in cash or in voluntary labor; hence price of the direct use and nonuse value irrigation should be increased to meet the conservation investment requirement. However, household living nearby the reservoir values the reservoir more compared to the distant users, which is mainly due to high benefits received from the tourism, such as wetland product consumption and tourism. After future use value, direct use value such as wetland goods and tourism remains the major contributor to total economic value of the reservoir followed by the indirect use value such as biodiversity conservation. The local government and other stakeholders should be sensitized for making investment for wetland restoration. In addition to this, tourism facilities and services need to be enhanced to increase motivation of the local people on wetland conservation along with generation local level employment opportunities. The sustainable financing strategy for the reservoir should be developed given the high economic importance of the wetland goods and services.

## Figures and Tables

**Table 1:** Sequential steps followed for total economic valuation.

Activities	Methods
Listing of use and nonuse value of wetlands	Observations, discussions with stakeholders and review of literature, local communities, rapid assessment
Prioritization of use and nonuse value	Scoring of goods and services by each category of stakeholders
Selection of valuation techniques	Review of previous studies and selection of the most appropriate and cost effective methods
Identify date needs	Development and preparation of survey instruments
Collection of data	Survey (household, tea-stall/restaurants)
Quantification of values & services	Analysis

Source: adapted from [13, 14].

**Table 2:** Types of use and nonuse value of reservoir and methods of valuation.

Value	Categories/type	Method of valuation	Source of data
Direct use	Wetland goods	Revealed price method	Household survey 2015
	Tourism	Tourism earnings (food,)	Survey of restaurants

Indirect use value	Water supply	Revealed price method	Water use fee collection
	Biodiversity conservation	Revealed valuation method (expenditure incurred by GON, conservation agencies)	[16], government agencies (2015)
	Carbon sequestration	Benefit transfer method	Carbon monitoring and carbon market studies

Nonuse value	Future use (existence & option value)	Contingent valuation methods/willingness to Pay	Household survey 2015

**Table 3:** Wetland goods and services from Jagadishpur Reservoir.

Value	Wetland goods and services	Prioritized goods and services
Direct use value	(1) Fish (2) Edible foods/fruits (3) Tortoise (4) Drift wood (5) Medicinal plants (6) Roofing materials (7) Wild birds/ducks (8) Tourism	(i) Wetland goods (fish, edible food/fruits, tortoise) (ii) Tourism

Indirect use value	(9) Grazing (10) Ground water recharge (11) Habitat conservation (12) Species conservation (13) Carbon sequestration (14) Irrigation (15) Religious (16) Cultural value (17) Livestock bathing (18) Private fish farming (19) Agriculture biodiversity improvement (20) Flood and landslide control	(i) Irrigation (ii) Carbon sequestration (iii) Biodiversity conservation

Option and existence value	(21) Educational purpose (22) Scientific research (23) Future use/protection (24) Prestige/Social pride	(i) Future use

**Table 4:** HHs consuming different wetland goods. Unit: % of HHs.

SN	Wetland goods	Adjoining area (*n* = 136)	Nearby area (*n* = 112)	Distant area (*n* = 136)	Overall (*n* = 384)
1	Local fish	43.4	17.9	16.2	26.3
2	Tortoise	0.7	0.0	0.0	0.3
3	Edible fruit	0.7	0.9	0.7	0.8
4	Drift wood	1.5	0.0	0.7	0.8

**Table 5:** Value of wetland goods consumed.

Area	Average value (NRs/HHs) (*a*)	Total HHs in JRRS (*b*)	Total goods consumed (*a∗b*)	% share by area
Adjoining	1640	6250	10,250,460	60.6
Nearby	713	5130	3,659,705	21.6
Distant	500	6010	3,005,000	17.8
Overall	*966*	*17390*	*16,915,165*	*100.0*

**Table 6:** Share of different value of wetland goods consumed.

SN	Wetland goods	Adjoining area (*n* = 136)	Nearby area (*n* = 112)	Distant area (*n* = 136)	Overall (*n* = 384)
1	Local fish	93.9	99.4	96.0	95.4
2	Tortoise	0.3			0.2
3	Edible fruit	4.3	0.6	1.5	3.0
4	Drift wood	1.6		2.6	1.4
	*Total*	*100.0*	*100.0*	*100.0*	*100.0*

**Table 7:** Value of recreation services.

Visitors	Number (*a*)^*∗*^	Average expenses (Rs) per visitor (*b*)^*∗∗*^	Total value (*a∗b*) (Rs 000)
Foreign	50	1270	63,500
SAARC	150	1823	273,450
Nepalese	10,000	874	8,740,000
*Total value *	*10,200*		*9,076,950*

Source: ^*∗*^IUCN [16]; ^*∗∗*^Tourism Entrepreneur Survey (2015).

**Table 8:** Value of water use.

SN	Wetland goods	Unit	Amount (NRs)
1	Irrigated area per season	NRs	6070
2	Irrigation use fee per season	NRs/ha	150
3	Total season	No	2
4	Water use fee (1*∗*2*∗*3)	NRs/year	1,821,000

**Table 9:** Value of carbon sequestration.

SN	Sources	Unit	Forest	Wetland	Remark
1	Forest	Ha	529	196	[16]
2	Annual carbon sequestration rate	tCha^−1^yr^−1^	1.38	1.3	[20, 21]
3	Annual total carbon sequestration (1 × 2)	tCyr^−1^	730.0	254.8	
4	Value of carbon	NRs^−1^tC	1064	1064	[24] (US$1 = NRs. 100)
5	Value of carbon sequestration (3 × 4)	NRs	776,741	271,107	
	*Total carbon sequestration*	*NRs*	*1,047,848*		

**Table 10:** HHs willing to pay for wetland conservation.

SN	Area	Willing to pay		Form of payment			
				In cash		In kind	
		Number	%	Number	%	Number	%
1	Adjoining area	103	75.7	100	97.1	103	100.0
2	Nearby area	74	66.1	73	98.6	74	100.0
3	Distant area	75	55.1	75	100.0	75	100.0
	*Total*	*252*	*65.6*	*248*	*98.4*	*252*	*100.0*

**Table 11:** Future use value of the reservoir.

SN	Area	Total HHs (*a*)	Willingness to pay (Rs/HH)			Total (NRs/HH) (*a∗b*)
			Cash	Kind	Total (*b*)	
1	Adjoining area	6,250	757	3335	4092	25,575,000
2	Nearby area	5,130	589	2614	3202	16,426,260
3	Distant area	6,010	279	1844	2124	12,765,240
4	*Total*	*17,390*	*539*	*2597*	*3135*	*54,517,650*

**Table 12:** Total economic value of goods and services.

Value	Good & service	Value (NRs)	Proportion (%)	Value per unit	
				NRs/HH	NRs/ha
Direct use	Wetland goods	16,915,165	17.9	973	863.0
	Tourism	9,076,950	9.6	522	463.1

Indirect use value	Carbon	1,047,848	1.1	60	53.5
	Biodiversity	11,200,000	11.8	644	571.4
	Irrigation	1,821,000	1.9	105	92.9

Nonuse value	Future use value	54,517,650	57.6	3135	2,781.5

	*Total value*	*94,578,613*	*100.0*	*5439*	*4,825.4*
